# The Recent Development of Acoustic Sensors as Effective Chemical Detecting Tools for Biological Cells and Their Bioactivities

**DOI:** 10.3390/molecules28124855

**Published:** 2023-06-19

**Authors:** Mostafa Gouda, Hesham S. Ghazzawy, Nashi Alqahtani, Xiaoli Li

**Affiliations:** 1 College of Biosystems Engineering and Food Science, Zhejiang University, 866 Yuhangtang Road, Hangzhou 310058, China; 2 Department of Nutrition & Food Science, National Research Centre, Dokki, Giza 12622, Egypt; 3 Date Palm Research Center of Excellence, King Faisal University, Al Ahsa 31982, Saudi Arabia; 4 Central Laboratory for Date Palm Research and Development, Agriculture Research Center, Giza 12511, Egypt

**Keywords:** acoustic sensors, quartz crystal microbalance, emerging technologies, piezoelectric materials, acoustic wave devices

## Abstract

One of the most significant developed technologies is the use of acoustic waves to determine the chemical structures of biological tissues and their bioactivities. In addition, the use of new acoustic techniques for in vivo visualizing and imaging of animal and plant cellular chemical compositions could significantly help pave the way toward advanced analytical technologies. For instance, acoustic wave sensors (AWSs) based on quartz crystal microbalance (QCM) were used to identify the aromas of fermenting tea such as linalool, geraniol, and trans-2-hexenal. Therefore, this review focuses on the use of advanced acoustic technologies for tracking the composition changes in plant and animal tissues. In addition, a few key configurations of the AWS sensors and their different wave pattern applications in biomedical and microfluidic media progress are discussed.

## 1. Introduction

The development of acoustic sensors is a very important scientific and technical issue. Acoustic sensors are widely applied in various technical systems that monitor the environment, provide biological and chemical safety, are used in signal processing devices, and numerous other applications. This technology is based on the use of acoustic wavenumbers, which has become one of the important emerging technologies [1,2].

Moreover, acoustic wave devices have been commercially used in many fields for several decays. Several of the emerging applications for acoustic wave devices as sensors include industrial and medical applications (such as tracking the industrial lines’ vapor, humidity, temperature, and product quality). For instance, super high sensitivity to humidity has been shown in sensors based on plate acoustic waves with graphene oxide sensitive film [3]. That is due to sensitivity of these sensors and intrinsic reliability. Virtually all acoustic wave devices and sensors are using piezoelectric materials to generate the acoustic wave. These piezoelectricity sensors are made of quartz materials that could have the resonator ability for stabilizing electronic oscillators [4]. Piezoelectricity refers to the production of electrical charges by the imposition of mechanical stress. Additionally, applying an appropriate electrical field to a piezoelectric material creates mechanical stress through an oscillating electric field, which propagates through the substrate and is then converted back to an electric field for measurement [5].

For example, the expression of AWSs occurs due to their detection mechanism based on the mechanical movements that cause acoustic waves to be considered and characterized [6]. These waves are moved on the surface of the material or through them. Any changes in the material characteristics could affect the velocity and/or amplitude of the hit acoustic waves and then could be sensitively detected and correlated to the corresponding physicochemical material measured [7,8].

This review provides the latest achievements related to the design, fabrication, modeling, testing, characterization, and advanced research trends in acoustic sensor technology as efficient analytical methods.

## 2. Operating Principles of Various Acoustic Sensor Types

The chemical sensors based on acoustic wave technology have continuously received research and technological attention [9,10]. Among the important advantages of the AWS sensors are their ultra-high sensitivity, excellent response time, small size, excellent selectivity, stability, and their compatibility with other emerging sensation technologies such as interdigital transducers (IDTs) [11]. The principle of sound radiation originated from vibrating plane surfaces (VPSs). The responsible detector can discriminate the generated harmonic transverse sound waves that move along an infinite plane surface in contact with a fluid to derive an expression for the associated acoustic radiation impedance [12]. These waves oscillate subsonically with wavenumbers higher than the acoustic wavenumber at the normally considered frequencies, but have only a very low extension into the fluid surface. On the other hand, waves traveling supersonically that have wavenumbers less than the normal acoustic wavenumber generate plane-traveling waves in the fluid that transport energy to infinite distances. This form of analysis can be extended to arbitrary distributions of plane surface vibration utilizing spatial wavenumber spectra for each component that represents the harmonic traveling waves of the studied samples. The sound fields generated by each wavenumber component are then pooled to provide the total radiated field characteristics. Additionally, this form of analysis has computational and interpretational advantages which are used to image the sources based on their reflected and radiated sound fields [13,14,15,16]. For example, Surface acoustic wave (SAW) and Bulk acoustic wave (BAW) are mainly used in the field of analytical measurements [17].

### 2.1. Surface Acoustic Wave (SAW) Sensors: Different Types of Devices

**Figure 1:**
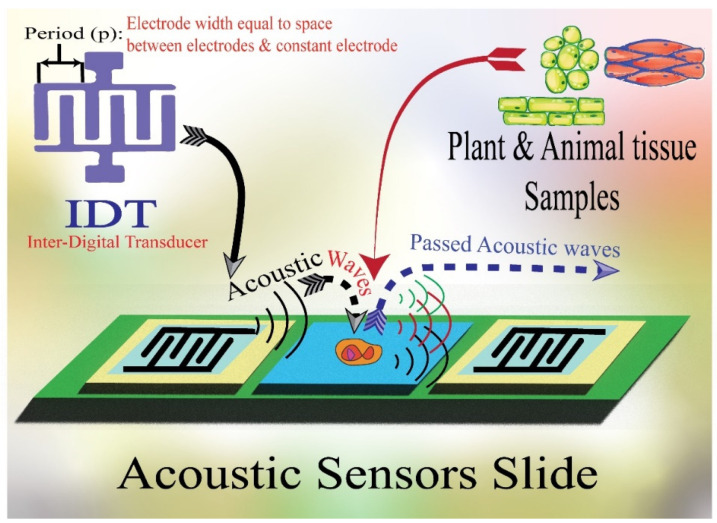
Schematics of SAW chemical sensors: a two-port delay line and a resonator with sensing overlayers for the target analyte.

Surface acoustic wave (SAW) is a specific kind of acoustic wave that travels down a material’s surface at a depth of roughly one or two wavelengths. Since the majority of the SAW energy is concentrated in the vicinity of the surface, the piezoelectric material’s surface is highly sensitive to even the smallest perturbations [18]. The intensities, phase angles, and output frequencies of the acoustic waves may change due to variations in wave velocity and attenuation brought on by acoustoelectric interactions or the mass loading effect, which can be observed using detection equipment [9]. Thus, these sensors are expected to fulfill the increasing demand for fast and sensitive detecting and monitoring technologies for various organic and inorganic materials (Figure 1). In addition, the most used elements in fabricating piezoelectric acoustic sensors are quartz, lithium niobate, gallium arsenide, silicon carbide, zinc oxide, aluminum nitride, graphene oxide, and lithium tantalate [3,19,20,21] (Table 1).

In addition, there are several SAW propagation types, such as Rayleigh waves, Sezawa waves, surface transverse waves, and shear horizontal modes. The sensor should have elements with high biological affinity such as gold (Au) to be adopted for the cell’s extra adhesion layer [32]. Each has specific advantages and disadvantages, which include cost, temperature dependence, attenuation, and propagation velocity. An interesting property of quartz is that it is possible to select the temperature dependence of the material by the cut angle and the wave propagation direction. Other materials with commercial potential include gallium arsenide, silicon carbide, zinc oxide, and aluminum nitride.

#### 2.1.1. Love Acoustic Wave Sensor Definition and Working Principle

**Figure 2:**
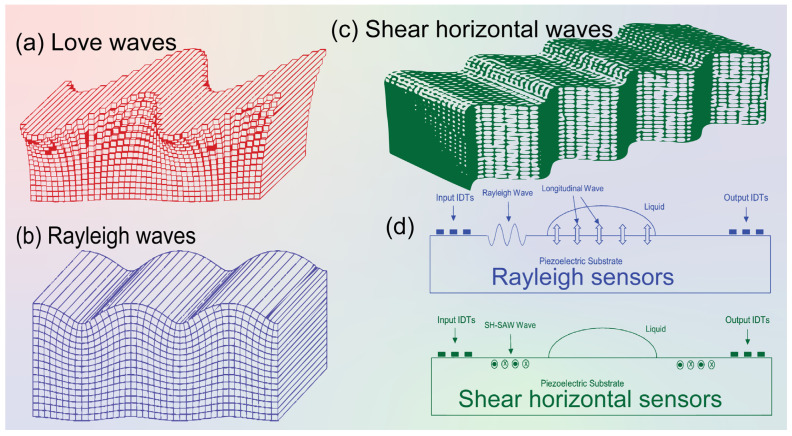
Schematics of different surface acoustic waves. (**a**) Love waves, (**b**) Rayleigh waves, (**c**) Shear horizontal (SH) waves [17,37]. (**d**) Schematic diagram showing Rayleigh wave and SH-SAW sensors’ application principles. The particle movement in and out is indicated by “cross: ×” and “bold dot: **•**” labels.

Love waves are surface waves with a horizontal motion that propagate shear mode waves supported on semi-infinite substrates with a waveguide layer that exhibits a shear acoustic speed lower than that of the substrate (Figure 2). It is well known that a Love wave has a pure shear horizontal polarization and a small attenuation of the wave is caused in liquid media. On the other hand, the other acoustic wave sensors utilize IDT to generate and detect propagating shear modes that polarized the surface acoustic waves and surface transverse waves. Love-mode acoustic devices are very promising as biosensors in gaseous and liquid environments because of their high sensitivity. For example, Du et al. [33] developed a Love-wave device based on SiO_2_/ST-cut quartz over a wide range of SiO_2_ thicknesses. In that study, the authors used devices with up to 7.3 μm thick SiO_2_, and they measured the mass sensitivity, velocity, insertion loss, oscillation frequency stability, and temperature coefficient of the frequency of the different functional layers. In addition, the authors reported that high sensitivity (≥300 cm^2^ g^−1^) can be achieved at a thickness between 3.5 and 6.5 μm of the quartz layer. Thus, numerical modeling of device thickness, mass, and liquid response should be specifically directed to a particular Love wave device type.

The Love wave sensor directly measures cell/substrate bonds via acoustic damping and provides 2D kinetic and affinity parameters. Other studies have applied the QCM sensor as a diagnostic tool for leukemia and, potentially, for chemotherapeutic agents. Acoustic sensors have also been used in the evaluation of the cytocompatibility of artificial surfaces and, in general, they have the potential to become powerful tools for even more diverse cellular analysis [25,34].

In another study of Love wave acoustic biosensors for monitoring the adhesion process of stem cells [23], the authors reported that Love wave biosensor is considered to be one of the most promising probing methods in biomedical research and diagnosis fields. It can detect the mechano-biological behavior of cells attached to the surface of the device. In that study, a lithium tantalate (LiTaO_3_)-based Love wave sensor was adopted as a cell-based biosensor to monitor the adhesion process. The effects of the viscoelastic cell layer and waveguiding layer on the propagation velocity *υ* and propagation loss (PL) were investigated. The resulting different storage and loss shear modulus revealed the potential of that technology’s usefulness in quantitative measures of cellular activities under multiple physiological conditions.

#### 2.1.2. Shear Horizontal Acoustic Wave Definition and Working Principle

Shear horizontal acoustic sensors are propagating modes involving the thickness of a thin piezoelectric plate and the detection and excitation by IDT. In 1968, Bleustein discovered shear horizontal surface acoustic waves (SH-SAW) based on a barium titanate (BaTiO_3_) piezoelectric material [35]. SH-SAW propagates due to the vertical movement of surface particles relative to the wave propagation direction and surface normality, as presented in Figure 2a. In terms of sensing capabilities, SH-SAW sensors are generally used for the detection of liquid samples due to less energy dissipating into the liquid during wave propagation, Figure 2d. In addition, there are many available materials for SH-SAW sensor substrates such as quartz, lithium tantalate (LiTaO_3_), and lithium niobate (LiNbO_3_), as well as other crystals such as langasite. The SH-modes in SH-APM resonators can be considered as superposition of plane waves with in-plane displacement reflected at a particular angle between the upper and lower face of the quartz resonator involving the full thickness of the resonator. This kind of sensor has very important applications in the analytical microbial pathogenicity and the responsible chemical measurements. For instance, Ji et al. [36] used the aptamer-based shear horizontal surface acoustic wave biosensor single-layered graphene film for high-sensitivity detection of the *Escherichia coli* cells endotoxin. These endotoxins are complex lipopolysaccharides that are produced from the cell walls of various Gram-negative bacteria (possessed lipid, core polysaccharide, and O-polysaccharide side chains), which are important molecules in measuring bacterial toxicity.

#### 2.1.3. Rayleigh Acoustic Wave Definition and Working Principle

Rayleigh waves are considered surface waves that travel only along a free surface or along the boundary between two dissimilar solid and liquid media (Figure 2). The name “Rayleigh” came from the British physicist Lord Rayleigh who discovered SAWs in 1885 [38]. Rayleigh waves are surface-normal waves, which makes them unsuitable for use in deep liquid sensing devices. Rayleigh waves are formed when the particle motion is a combination of both longitudinal and transverse vibration giving rise to an elliptical retrograde motion in the vertical plane along the direction of travel. This technology has a vast application for the biochemical analyses of biological fluids. For example, Agostini et al. [31] used a label-free sub-nanomolar Rayleigh surface acoustic-wave (R-SAW)-based biosensor in demonstrating the biomolecular detection of dried liquid solids. That biosensor integrated two interdigital transducers for positive and negative reflectors. The experiments demonstrate a limit of detection of 104 pM and a normalized sensitivity of −296 m^2^ kg^−1^. In comparison with similar acoustic-wave-based systems, both the sensitivity and limit of detection of that sensor were higher than those of Love-SAW biosensors in its application in cancer biomarker detection. Additionally, the velocity of propagation of any body wave in any homogeneous, isotropic material is determined by the elastic moduli and densities of the material through which it passes. The traditional seismic survey uses only compressional waves due to easy detection of the vertical ground motion in the detectors that becomes fast because of high-speed wave velocity. On the other hand, the recording of stress and Rayleigh surface waves provides greater information about the subsurface, but at a cost of greater data acquisition and consequent complex processing [22].

#### 2.1.4. Quartz Crystal Microbalance (QCM) Definition and Principle

Quartz crystal microbalances are suitable transducers for chemical and biochemical sensing [28]. They are used to detect the micro mass changes and physical properties of thin layers deposited on the crystal surfaces and are capable of real-time detection [39]. These devices are using silicon dioxide (SiO_2_) known as quartz and have higher shear wave velocity and higher density than polymer materials such as polymethylmethacrylate (PMMA), providing the potential for lower acoustic loss [40]. Due to its simplicity, small volumes of samples, absence of expensive reagents, easy fluid control in the dynamic mode, and low cost, it has been reported as important for detection of dynamic biomolecules.

Several studies have reported on QCM biosensors as being an alternative method to conventional analytical methods such as chromatography and immunosorbent assays [22]. One of the important advantages of using these sensors is that they are reusable, with high sensitivity. Matatagui et al. [22] developed an immunosensor based on a QCM device with very high sensitivity for the detection of antigens in real-time status. In that study, the QCM immunosensor was well-established for the detection of immunological reactions. The biosensor comprises a quartz crystal with an antigen or antibody immobilized on its surface. The reaction between antigen and antibody is thus promoted due to the antigens being transported by the movement of the liquid medium. Moreover, immunoreactions occur due to diffusion of the antigens.

#### 2.1.5. Sezawa and Leaky Pseudo-Acoustic Mode Definition and Principle

From all the studied types of acoustic waves, Sezawa SAW has high acoustic velocity and a large electromechanical coupling coefficient that make it useful for elucidating the bio-sensing properties [41]. It has the highest resonant frequency of up to 17.7 GHz and a signal amplitude of 20 dB with an electromechanical coefficient equal to 0.92% [42]. These devices have shown promising characteristics in many applications due to their compact structures, low power consumption, easy construction and simple packaging, high sensitivity, and fast response that can be used for diverse applications such as sensing ultraviolet radiation, gas, humidity, pH, and biomolecules. For instance, Kuznetsova et al. [43] mentioned that the Sezawa wave can be used to develop highly sensitive humidity acoustic sensors. On the other hand, Suenaga et al. [44] mentioned that leaky pseudo-SAW (LPSAW) is a high-order mode that radiates its acoustic energy into both the water and the substrate. In addition, the water medium is assumed to be an ideal liquid for this mode of measurement.

### 2.2. Bulk Acoustic Wave Sensor Definitions and Working Principles

The basic design of a BAW resonator consists of a piezoelectric material sandwiched between two metal electrodes. It works on the inverse piezoelectric effect when the electric field is applied across the metal electrode, thereby exciting the acoustic wave in the direction of thickness and vice versa. Zou et al. [45] studied the potential application of thin-film bulk acoustic wave resonators and vigorously supported aluminum nitride (AlN) as a piezoelectric device. In that study, the authors were able to achieve high-quality factor (QF) by reflecting the bulk acoustic wave with air interface at the bottom and top surfaces of resonators. Bulk acoustic waves propagate in a vertical direction with a high-frequency resonance signal, in which different materials and structures of BAW are utilized for optimizing the resonance of acoustic waves between the top and bottom electrodes [46]. Gomes [47] reported that BAW devices have been developed for the quantification detection of a large number of compounds, such as organic compounds, pollution, and biomarkers [48].

## 3. A Comparative Analysis between BAW and SAW

**Figure 3:**
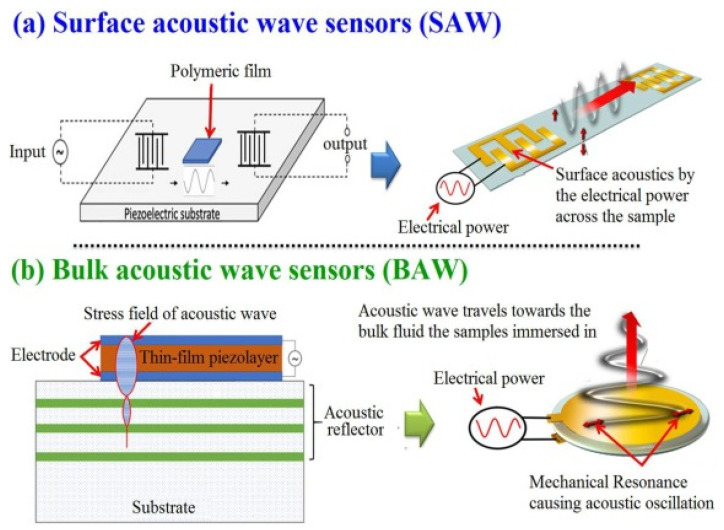
Graphic depicting in general terms the processes for the generation of surface and bulk acoustic waves (open access free permission) [50].

As SAW was invented almost four decades before BAW devices, both of these sensors have a multitude of measurements in physical, chemical, and biological fields [49]. Both of these sensors are mainly based on oxide ceramics and metals such as quartz. Their output signals such as frequency and phase lend themselves well to digital measurement; they are typically operated at high frequencies [17]. The use of SAW and BAW for chemical composition measurement to draw chemical images of animal and plant cells and tissues and visualize their biomolecules has become one of the hot scientific research areas [50]. An acoustic wave sensor typically consists of a piezoelectric substrate coated with sensing material (polymeric film), and input and output transducers are commonly used for chemical composition purposes. The difference between SAW and BAW is based on the acoustic wave propagation direction: if it moves on the surface of the substrate, it is called SAW, while if the wave propagates through the substrate, it is called BAW (Figure 3).

## 4. Characterization of Electrophysical Properties of Acoustic Sensors

**Figure 4:**
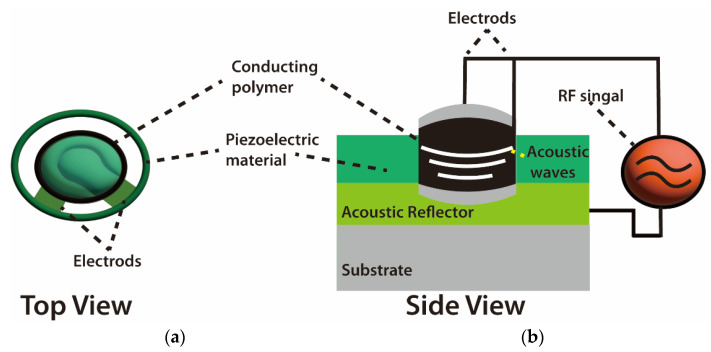
Schematics of bulk acoustic wave (BAW) chemical sensors. (**a**) Vertical top view. (**b**) Side view of BAW sensor system [54,55].

The mode of wave propagation by a piezoelectric substrate is used to characterize acoustic wave devices. The majority of the energy density is contained in the vicinity of the surface due to the order of wavelength penetration depth of these waves [51]. Because of this, any physical or chemical changes on or near the surface causes the waves to alter, and, consequently, the devices that rely on them [52]. For instance, Kiontke et al. [53] used SAW nebulization assistance in substantial signal enhancement. In that study, the authors mentioned that SAW increased the sensitivity response of aminophenols and phenylenediamines up to eight times without any heating for the studied sample due to the increase in the accessible droplet surface area. In addition, the working frequency of SAW devices can be adjusted across a large range (from MHz to GHz), making it possible to fine-tune their sensitivity and use them wirelessly. Additionally, based on the material and boundary conditions, many combinations of velocities and displacement directions can be used to distinguish acoustic waves. As another characteristic example, bulk waves are waves that propagate through the substrate [54]. The thickness shear mode (TSM) resonator and the shear horizontal acoustic plate mode (SH-APM) sensor are the two most widely utilized bulk acoustic wave (BAW) devices (Figure 4).

## 5. Design of Various Types of Acoustic Sensors

The design of the used ASW is based on the used samples’ characteristics and nature. For instance, SAW sensors could typically run between 25 and 500 MHz of the acoustic wave frequencies [56]. The surface wave is excessively attenuated when liquid comes in contact with a SAW sensor because of the ensuing compressional waves. The sensitivity of the sensor is often inversely correlated with the energy perturbing the propagation channel [57]. Meanwhile, the energy is often transferred from one surface to the other surface by way of the bulk material in bulk acoustic wave sensors [54]. In addition, the energy density on the surface, which is where the sensing is carried out, is reduced by this energy dispersal. Moreover, other design considerations when selecting acoustic wave sensors include oscillator stability and noise level [57]. These sensors are capable of identifying and detecting substances at ppb levels of concentration [58]. The piezoelectric transducer characteristics, the center frequency, the sensor layer characteristics (such as material qualities, thickness, and surface roughness), and the operating temperature all affect sensitivity, which can be measured in Hz/ppm or Hz/% [59]. The mass sensitivity (f/m) of these characteristics has been demonstrated in numerous tests to rise the root mean square (RMS) of the operating frequency that enhances the signal sensitivity. In ideal circumstances, sensitivity rises with layer thickness; however, variations in a layer’s roughness, crystallinity, and hardness with layer thickness could have an impact on sensitivity [32]. Nevertheless, the vast majority of acoustic sensors are Si-based products, which means that they lack several essential elements for the biological sample, including macro- and microelements, such as essential amino acids and fatty acids. Therefore, the acoustic sensor is a great vehicle for fortification with highly valued technological ingredients. For instance, Jiang et al. [60] used SAW sensors with Love acoustic waves by employing SiO_2_-coated piezoelectric for detecting hemagglutinin (HA) antibodies that related to Influenza antigen detection with appropriate surface functionalization. The authors mentioned that the HA detection limit concentration is as low as 1 ng mL^−1^.

**Figure 5:**
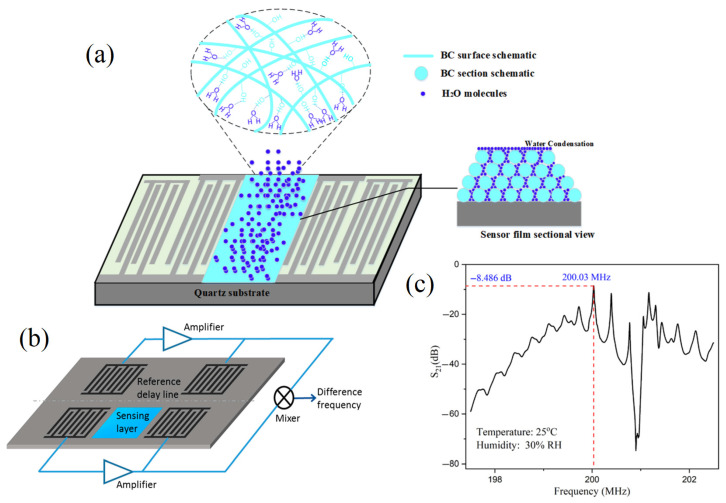
Schematic of a dual delay line SAW sensor for compensation of thermal and humidity drifts. (**a**) SAW by using the Bacterial cellulose (BC) mode of action. (**b**) The sensation layer and reference layer complete SAW system. (**c**) The potential obtained spectrum from using different acoustic frequencies [54,58,62] (copyright permission no: 6895891235874).

The adequate enrichment of acoustic sensors is more effective to improve their detection sensitivity. In this context, chemical analysis technologies should serve with the technological studies to achieve the goal of adding the beneficial essential elements to improve the stability and accuracy of the acoustic sensor for enhancing its feasibility as a worldwide distributed functional chemical analysis technology [61]. For example, the acoustic sensor enriched with powder bacterial cellulose showed benefits in many aspects such as high-sensitivity characteristics [62]. Meanwhile, SAW torque sensors are utilized in practical applications with their centerlines at right angles and their sensitivity to temperature drift; the mass loads make several applications possible for these kinds of sensors, such as their film thickness sensors and particle sensors. The sensor transforms into a particulate sensor if it is covered with an adhesive substance; any particle that lands on the surface stays there and disrupts wave propagation. A 200 MHz ST-cut quartz SAW has been reported to have a mass resolution of 3 pg, which is 1000 times more sensitive than the tested 10 MHz TSM resonator. Wang, Guo, Li, Long, Tang, Zu, Ma, Du, Tang, Torun and Fu [62] developed a SAW for tracking the media humidity as a sensor based on bacterial cellulose (BC) coated by quartz. The authors mentioned that the BC-SAW sensor exhibited good short-term repeatability and long-term stability for the media humidity sensation (Figure 5).

## 6. Application of Acoustic Sensors in Biochemical Material Detection

**Figure 6:**
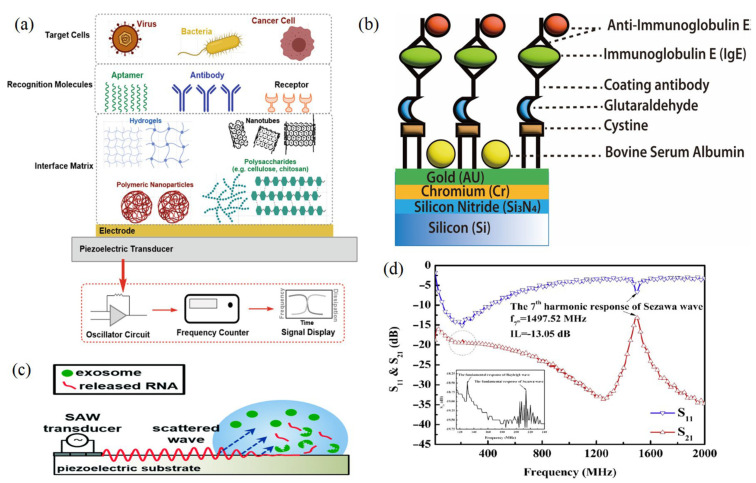
(**a**) Components of an acoustic biosensor to capture whole cells. (**b**) Schematic diagram for the SAW sensor integration of cystamine, glutaraldehyde, and IgE antibody/antigen multilayer. (**c**) Schematic of SAW-based lysing of exosomes to release RNA for detection [24]. (**d**) Frequency response of the Sezawa-mode SAW device [32,68] (copyright permission no: 5543441314248).

The application of acoustic wave sensors for monitoring the frequency variations in waves that pass through biochemical materials makes it a perfect technology for identifying the biomarker compounds such as lipids and proteins (Table 1). For instance, it can identify cancer proteins that are bound to a sensor surface receptor [18] (Figure 6a). The piezoelectric effect, a phenomenon where an initial electric signal is transformed into a mechanical displacement, is what causes the initial wave to be produced. This movement is transmitted through the crystal as a wave. In addition, the signal in the SAW sensor moves through the material from the input transducer to the output transducer, where it is transformed back into an electrical signal. According to the Center for Nanoscale Materials (Zhejiang University, China), the wave’s frequency is dictated by the sound wave’s speed through the material. The capacity of researchers to identify frequency or variations in the waves as they spread is what makes these gadgets effective as sensors [63]. The attachment of chemicals to crystal receptors or proteins to antigens results in changes in the density of the crystalline medium, which in turn induces variations in pitch are an important factor to be considered related to this technology. According to Mandal and Banerjee [64], when something binds to the acoustic sensing layer, the wave characteristics change, then the detector can measure those changes. These novel sensor theories have several advantages, one of which is the possibility of making them battery-operated and portable. However, in order to do that, researchers must figure out how to use less energy to run the apparatus. The ability to run with very low power consumption is necessary to make something portable, according to Sankaranarayanan [57]. Due to the matrix crystal characteristics, the first generation of SAW sensors lost a significant portion of their signal inputs. To solve this issue, Sankaranarayanan and his colleagues inserted zinc oxide-filled microcavities that behave like bumpers in a bowling alley by trapping energy near the surface that would otherwise be lost to bulk waves [65,66]. The microcavities, according to Laidoudi et al. [67], cut energy losses by 50%. This means that we are a great deal closer to producing these portable biosensors.

### 6.1. Micromolecular Chemical Analyses by Acoustic Sensors

The natural phytochemical detection by acoustic sensor and phytochemical potential bioactivities for human health led the scientific community to examine the acoustic sensor from a new scientific perspective in terms of their potential uses in facing the demands of the national and international chemical problems such as the hazardous use of the organic solvents and heavy elements. For instance, acoustic sensor products are the least expensive chemical analysis commodity, especially in large-scale applications. Tess and Cox [69] mentioned that the acoustic sensor can serve as a technological source for tracking cellular amino acids. Moreover, the acoustic sensor amin structures have unique functional and technological properties to be used in chemical analysis applications [70].

**Figure 7:**
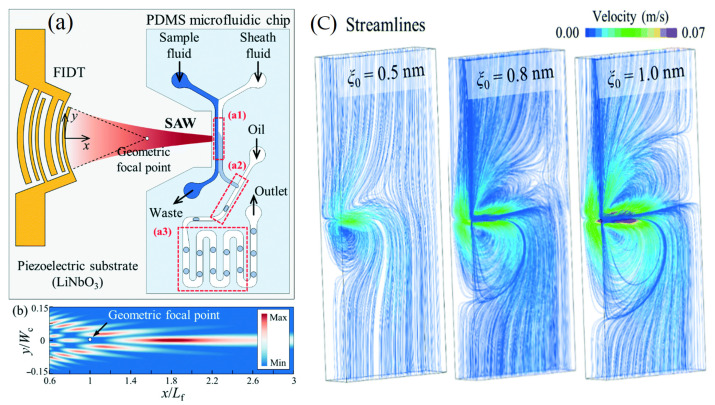
(**a**) Schematic diagram of the acoustofluidic device for the production of droplets with fine-tuned chemical concentrations. (**b**) Numerical simulation of the surface acoustic wave amplitude field for the transducer based on the exact angular spectrum of plane wave theory. (**c**) Variations in the solute concentration with the duty cycle (3.33 to 30%) and the amplitude of the applied voltage (2.23, 2.47, and 2.76 VPP) [18] (copyright permission no: 4859961463671).

Researchers have tested several macro- and micromolecular chemical analyses by acoustic sensors combined with other optical techniques to enhance the chemical, functional, and technological properties of chemical analysis and their final collected results. In addition, acoustic sensors can solve traditional technologies such as the dye interference of spectroscopy [1]. As an example, SAW is well known as an excellent choice for deficient protein samples. Recently, several studies recommended the use of a SAW compared to other ASWs due to its pharmaceutical and nutraceutical application high sensitivity due to the increase in the surface area to mass ratio. Therefore, membrane-based systems are promising concerning their sensitivity [71]. In addition, many chemicals were studied using different acoustic intensities of the acoustic transducers which have the potential to select the exact chemical molecule fingerprint [18,72] (Figure 7).

Bourdeau et al. [27] developed an acoustic method for in vivo visualizing and imaging of the microbial cellular chemical composition inside the mammalian hosts. This method proved its efficiency in its application as an analytical method. For example, acoustic sensors based on quartz crystal microbalance (QCM) were used to detect tea aroma (e.g., linalool, geraniol, linalool oxide, and Trans-2-hexenal) during its fermentation process [28]. In addition, recent emerging and chemical-free technologies related to in situ detection of the physicochemical changes in the biological media are important for studying the cellular-based levels and the functional components concentrations, such as Raman, circular dichroism (CD), and nuclear magnetic resonance (NMR) [73,74]. Several studies have documented the efficacy of these technologies for the replacement, enhancement, and improvement of various conventional analytical techniques in detecting animal and plant tissues [75,76,77,78,79,80]. On the other hand, these methods have common linked challenges, such as the fluorescence dye intervention on the used photonic optical sensors. As a proper solution, Garrett and Wang [21] reported that optical acoustic sensors could achieve high levels in biochemical photoacoustic imaging of biological systems. In addition, Westerveld et al. [81] fabricated an optoacoustic imaging system for the mouse brain tissue using >15 MHz acoustical frequency and <100 μm wavelength in water. This tomographic imaging relies on a low detection limit (noise/pressure, NEP); it was mentioned that piezoelectric sensors rely on their mechanical resonance to enhance the signal amplitude. In addition, Tian et al. [82] developed an acoustic phononic crystals method to support the acoustic topological states with complex wavenumbers that can configure the formation of rainbow edge waves of the studied samples. This study may spark future investigations of topological states with complex wavenumbers in the graded materials.

### 6.2. Application of Acoustic Sensors in Protein, Lipid and Biomarker Level Detection

**Figure 8:**
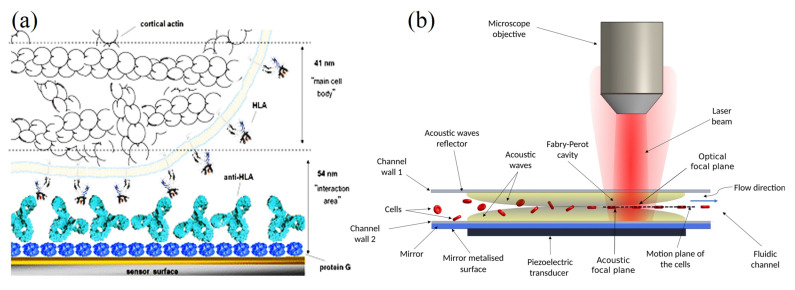
(**a**) Schematic illustration of the acoustic sensor interface for cells interacting via membrane HLA immunoglobulin molecule with surface-immobilized antibodies. The dashed lines for penetration depths of the quartz crystal microbalance (QCM) sensor with 95 nm. (**b**) Microfluidic channel producing acoustic manipulation. (**b**) The cells’ microfluidic channel serves as an acoustic reflector sandwich between the wall and the piezoelectric transducer to complete the interferometer through the acoustic waves (yellow) [94,95] (copyright permission no: 600121448).

The application of acoustic biosensors as alternative methods for protein and their functional impact analysis has become a popular scientific area [22]. Cho et al. [83] studied the lipid–protein interactions and protein–protein interactions of the cellular membrane by using a QCM biosensor. They reported that QCM could detect the charged zwitterionic functional impact of the tissue lipid bilayer compositions. In addition, Jiang et al. [84] employed acoustic sensors for in vivo imaging of wild plants and in vitro cell imaging using quantum dots technology to bio-image the chemical composition of plants (Figure 8). That could be obtained through the ability of these biosensors for detecting the functional groups of proteins in the biological fluids. For instance, the covalent immobilization of urinary proteins allowed the selective detection of nitroaromatic compounds which may occur in explosives [85]. For instance, Pomowski, Baricham, Rapp, Matern and Lange [30] used SH-SAW coated with 2-methacryloyloxyethyl phosphorylcholine polymer for the label-free detection of the inflammatory marker C-reactive protein in human serum. The authors mentioned that SH-SAW allowed significant differentiation between human CRP serum concentrations lower than 10 mg L^−1^ which could effectively diagnose the bacterial infection. In addition, these sensors have been recently developed for biosensation and detection of SARS-CoV-2-related antibodies. In a study by Peng et al. [86], it was mentioned that SH-SAW achieved high correlation coefficients (*R* = 0.99) at different concentrations (34.375–1100 ng mL^−1^) of its protein antibodies, with better sensitivity compared to ELISA.

Acoustic Love waveguide sensors are potential effective tools for detecting the lipid mono- and bilayers through the use of a thiol-coated surface area. The sensitivity of AWS revealed that it could detect the lipid layer mass change during layer deposition and the viscoelastic properties of the interface could change significantly [87].

Regarding the importance of AWS in the biomarker field that is related to the global burden, Onen et al. [88] fabricated a urinary SH-SAW technique for detecting the anti-apoptotic protein of the B-cell lymphoma 2 (Bcl-2) for ovarian cancer early detection. In that study, the sensor was able to successfully detect Bcl-2 in the concentration range of 0.5 to 12 ng mL^−1^. Thus, it could be applied as a promising technology in the diagnosis and quantification of ovarian cancer. The sensors detect cells mostly via their sensitivity in viscoelasticity and mechanical properties. In particular, the QCM sensor detects cytoskeletal rearrangements caused by specific drugs affecting either actin microfilaments or microtubules.

#### Acoustic-Based Biosensors for Bio-Imaging the Live Cell Enzymes and Active Ingredients

The use of acoustic sensors for the biochemical analysis of live cells and particles is an emerging technology that integrates acoustics and microfluidics [89]. In the last decade, this technology has attracted significant attention due to its biocompatible, contactless, and label-free nature. For instance, it has been widely validated in the separation of cells, viruses, biomolecules, exosomes, and submicron bioparticles [90].

To increase the detection sensitivity, micro/nano-acoustic biosensors are typically employed to increase the activity of particular biomolecules such as enzymes [91]. These biosensors are built on a special kind of gas vesicles, which are protein nanostructures packed with air that vibrate in response to ultrasound vibrations. For example, the incorporation of acoustic biosensors markedly increased the efficiency of single-cell enzyme activities. This was proved by Lakshmanan et al. [92] who studied the physicochemical characteristics of the bacterial cell-released bioactive enzymes using an acoustic biosensor of endopeptidase. The authors claimed that acoustic sensors at 132 and 477 kPa levels of waves showed high resolution for tracking the single-cell proteases.

Deep tissue can be easily imaged with high spatiotemporal resolution using acoustic waves. In the works of Jiang et al. [84] and Jiang et al. [8], application of quantum dots technology for in vitro cell imaging and in vivo imaging of natural plants allowed for the bio-imaging of plant chemical composition. Moreover, the acoustic biosensors were employed by Lakshmanan, Jin, Nety, Sawyer, Lee-Gosselin, Malounda, Swift, Maresca, and Shapiro [2] to image the activity of enzymes within the mouse gastrointestinal tract. In addition, Barie and Rapp [93] used an XY-cut LiTaO_3_ SH-wave sensor at 380 MHz acoustic resonance for detecting glucose oxidase (GOD) enzyme. In that study, the authors used antibodies with high specificity that can bind up to 16 ng/mm^2^ of protein with a high sensitivity detection limit (59 Hz/ng).

The idea behind using acoustic-based biosensors is to couple the measurement process, such as analyte adsorption, with a change in the acoustic wave’s physical characteristics, such as its frequency and velocity, which could be related to the analyte concentration [91]. Because of light scattering and interference with their phytochemicals’ fluorescents, existing molecular biosensors based on fluorescent emission are of limited use.

### 6.3. Application of Acoustic Sensor Detection of Single Cell Metal Elements

**Figure 9:**
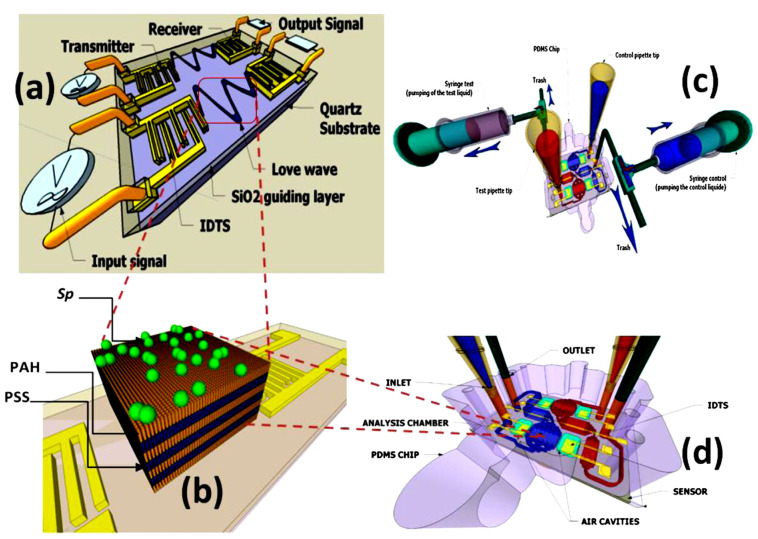
A technological study of the impact of acoustic sensor acoustic sensors on the microalgae chemical composition. Schematic of a SAW sensor with a hybrid biofilm of polyelectrolyte microalgae. (**a**) Scheme of SAW. (**b**) *Spirulina* immobilization on a polyelectrolyte multilayer (PEM) coated with a layer by layer (LBL) method. (**c**,**d**) hydrodynamic chip with microfluidic network, aligned on SAW [25,34] (open access permission).

The use of acoustic waves for studying the cellular heavy metal and their interactions with the cell’s DNA has been recently established. For example, Beabout et al. [96] used An Echo acoustic transducer for testing the ability of combined sensor reactions to detect multiple heavy metals when the arsenic, cadmium, and mercury DNA circuits are combined into one reaction at a 10 μM limit of detection. In addition, there are several studies on using acoustic sensors for tracking microalgae single-cell elements. Studies showed a strong relationship between acoustic sensor types and their impacts on microalgae chemical composition studies [25,34] (Figure 9). The results of these studies provided an insight into the different interactions of dried biomass of Spirulina (*A. platensis*) with metallic cations using acoustic and microscopic tools. The authors applied an acoustic wave platform to perform real-time monitoring of the interaction of a heavy metal solution in contact with Spirulina cells. Love wave and shear horizontal wave sensors were used, and the authors reported that Love wave sensors were ideally suited for (bio)chemical applications in gases and liquids [40]. In addition, this technique has reached high sensitivity for the characterization of heavy metals at low concentrations (10^−12^ M). Gongi et al. [97] characterize the cadmium (Cd^2+^) and mercury (Hg^2+^) heavy metal ions based on the extracellular polymeric substances (EPSs) isolated from a Tunisian thermophilic microalga strain *Graesiella* sp. In that study, the authors used quartz-based AWS and a 1.2 × 1.2 cm^2^ active area with an acoustic resonance of 118 MHz. It was mentioned that Love waves sensors showed good analytical performance and a low detection limit of 10^−10^ M. This could be due to the structural complexity containing hydrophilic and hydrophobic groups of the microalgae EPSs that can absorb and retain the water molecule, which offers them gelling characteristics and increases their ability to interact and adsorb the heavy metals. Additionally, Jiang, Jin and Gui [84] utilized an acoustic-assisted solvothermal process for quantum dot-based bio-imaging of plant zinc ion. The technique’s feasibility, according to the authors, might be employed for both in vivo and in vitro imaging of real plants [94,95].

### 6.4. Acoustic Based Sensors for Cell-Level Detection

A SAW chemical sensor’s reaction time has been demonstrated to be temperature-dependent [98]. The rate at which the analytes diffuse or dissociate may increase as the temperature rises, reducing the response time. For instance, Chen, Chang, Cheng, Shen and Kao [32] reported that the usage of an immobilized SAW sensor with an isolated cavity to measure IgE antibody sensitivity reached 4.44 × 10^6^ cm^2^/g. This should have a special condition for the used substrates and conditions for increasing the detection rate of the allergens in the biological fluids and tissues based on the Sezawa acoustic wave frequencies (Figure 6). Furthermore, all sonic wave sensors are sensitive to changes in a wide range of physical characteristics [99]. Many factors influence the rate of layer–analyte interaction and, consequently, the response time of a SAW sensor [100]. In the case of mass-based sensing layers, the rate of diffusion of the adsorbed mass into the film, to the piezoelectric substrate, and back to the film surface heavily influences the response and recovery times of a SAW sensor [101].

It is known that every organism either consists of cells or by itself is a single cell, whether it is an animal, a plant, or a microorganism. Therefore, it is very important to understand the cellular behavior in both biological and biomedical fields by studying the cells’ chemical compositions or their interactions in the surrounding environments. For instance, several studies used acoustic sensors for studying cellular activities such as adhesion activity, which is considered one of the most important cell functionality-related biomarkers that are affected by their signaling pathways that direct cell functions [23].

Meanwhile, acoustic biosensors offer the possibility to analyze cell attachment and spreading. This is due to the offered speed of detection, the real-time non-invasive approach, and their high sensitivity not only to mass coupling but also to viscoelastic changes occurring close to the sensor surface. QCM and surface acoustic wave (Love wave) systems have been used to monitor the adhesion of animal cells to various surfaces and record the behavior of cell layers under various conditions [94]. In addition, Mejia Morales, Glynne-Jones, Vassalli and Lippi [95] have established an acoustofuidic interferometric device for high-throughput monitoring analysis of the microalgae cells (*Tetraselmis* sp.) and yeast (*Saccharomyces cerevisiae*) cells based on their physical properties (such as morphology or mechanics). In that method, the authors established a flowing channel with fundamental acoustic mode resonance (6.682 MHz) for passing the cells through it. In addition, the authors identified two types of cell-induced perturbations (strong and weak). The first occurs when the cell crosses the optical resonator’s axis, while the second takes place when the cell crosses any other portion of the resonator. As mentioned, that method provided high sensitivity and a speed potentially suitable to obtain the high throughput necessary to handle the variability stemming from the biological diversity of the cells (Figure 8b) [95].

The use of AWS can qualitatively monitor the dynamics and counting the varied cell types by relying on an acoustic wave which penetrated into the basal plane region of the cell. Qualitative data about the cell count and dynamics can be provided based on the used acoustic frequency [102]. A QCM cell composed of a cell culture incubator and a detection system was developed to monitor the growth of epithelial colorectal adenocarcinoma cells (Caco-2). That fabricated system allowed investigations of count for the proliferated and dead cells [103].

### 6.5. Acoustic-Based Sensors for Monitoring Cell Culture Environment

**Figure 10:**
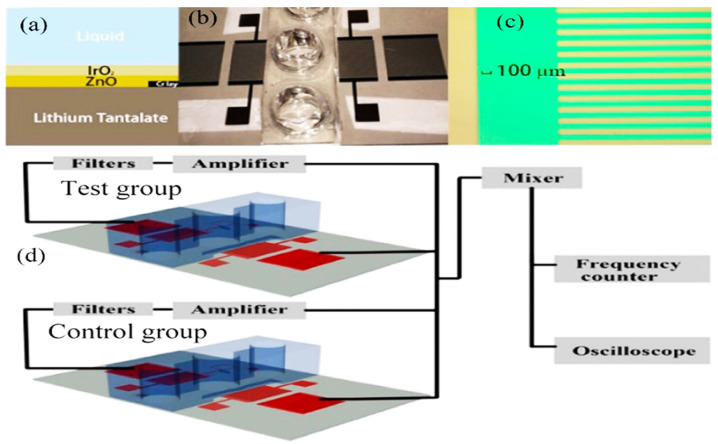
(**a**) Double-layer construction SAW sensor. (**b**) Fabricated resonator and fluidic well. (**c**) Microscopic image of the finger pairs. (**d**) Potential experimental setup illustration [108] (copyright permission: 5567480548369).

A fundamental variable in a culture medium such as pH, glucose, CO_2_, and sucrose could negatively affect the cells during their cultivation processes. For example, the biological processes are exquisitely sensitive to acid-base chemistry including the concentration of protons H^+^ and carbonate (CO_3_^2−^) formation. These conditions should be real-time monitored by advanced sensors for controlling them by an appropriately formulated buffering regime such as CO_2_/HCO_3_^−^ [104]. For instance, Wang et al. [105] monitored the pH condition of the tumoroid cultures by SAW sensors. They fabricated novel acoustic pH sensor based on LiTaO_3_ to generate a 13.91 MHz center frequency for tracking the tumor cell (tumoroid) culture pH (Figure 10). They tracked the pH changes during 5 days of cultivation. The principle of using QCM for pH detection is based on the pH reactive polymer layer to measure the polymer mass loading by shrinkage and swell. This action causes transition function of electrical corrosion between the two oxidation states [106,107].

### 6.6. Acoustic Sensors for Cancer and Tumor-Level Detection and Tumoroid Cultures

**Figure 11:**
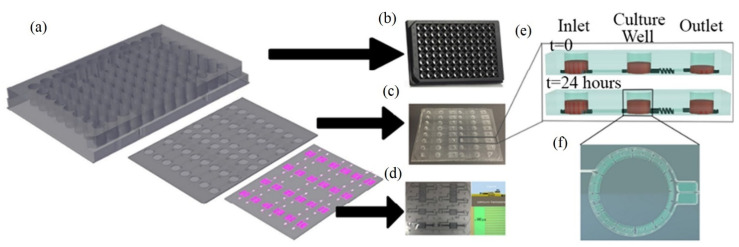
Design of the SAW-based gravitational microfluidic system. (**a**) Microfluidic 96-well plate with integrated sensor layer at the bottom. (**b**) Bottomless 96-well plate. (**c**) Microfluidic channel layer. (**d**) Surface-acoustic-wave (SAW)-based sensor layer. (**e**) 3D vertical image of the well fluid levels at 0 h and after 24 h. (**f**) A real image horizontal view of the culture well (copyright permission: 5567500483871).

The application of acoustic sensors, especially SAW, for differentiating the healthy cells from the aggressive and nonaggressive tumor cells and monitoring the cancer cell activities has emerged as a new significant technology [102]. This technology is among the key technologies for tumor and cancer biomarker detection, in which its frequency sensitivity could reach 8.704 pg/Hz with a mass sensitivity of 2810.25 m^2^/kg for detecting mammoglobin cancer-related antigens [109]. Zhang et al. [110] used a recyclable chitosan-based QCM biosensor for real-time detection of breast cancer cells. The authors compared human erythrocyte, endothelial cell, and oral epithelial cells by using a 5 MHz acoustic resonance. The obtained results showed that QCM successfully discriminated the different cell types with a wide linear range of 4.5 × 10^2^–1.01 × 10^5^ cells/mL and a detection limit of 430 cells/mL. Hianik [108] reported the effectiveness of the fabricated immunoacoustic biosensor for diagnostics of leukemia blood cell cancer. For that purpose, the sensor operates at frequencies of around 100 MHz. In another study by Hao et al. [111], the authors established an immunoacoustic biosensor for diagnostics of leukemia (blood cell cancer). That applied study used SH-SAW for the detection and separation of Jurkat and K562 leukemic cells by using a 122.5 MHz frequency. The authors mentioned that maximal sensitivity of detection was 10^3^ Jurkat cells/mL after a 15 min detection time. That method successfully differentiated the cells in a mixture of Jurkat/K562 cells (1:1000) at a 10^6^ cells/mL concentration. Meanwhile, SAW has been used in the field of tumoroid culture detection. Wang et al. [112] designed a SAW system for drug sensitivity assay of the colorectal perfused tumoroid cultures (Figure 11). That study was focused on the tumoroid cell proliferation, density and pH. During the experiments, the wells mentioned in Figure 11 were filled with the tumor cells and culture medium, and the control group was filled with blank culture medium. The principal theory, when the SAWs propagate through the detection area where the nanofiber scaffold was attached, the phase velocity was changed due to the mass loading changes caused by tumor cell growth. As the tumoroids grow, the frequency and phase for the test group sensor changes while the frequency of the control group sensor remains nearly constant. In addition, when the acoustic waves travel through the well that has pH electrodes, the potential difference is generated between the two electrodes located on the bottom and top of the two sensor layers. The potential difference is affected by the conductivity and dielectric properties of the liquid and electrode layers. As a result, an impulse signal to the input interdigital transducers was generated that could distinguish the cell densities and the pH calculations.

### 6.7. Application of Acoustic Sensors in Biological Fluid-Level Detection

The AWS has the ability to detect chemicals of the biological fluids and liquids. This could be due to the fact that the AWS device waves have poor performance in liquids as the propagating wave’s vertical component is blocked by the liquid. Additionally, for liquid sensing, the Love wave acoustic sensor has the maximum sensitivity for this purpose [113]. Vellekoop [114] reported that the ST-quartz shear horizontal (SH) acoustic plate mode (APM) sensor can distribute throughout the bulk of the substrate that is developed for sensing liquid particle displacements. The shear wave transmits in the low shear acoustic velocity material (upper layer) with a higher shear acoustic velocity, thus representing bilayer geometry. At a specific frequency, the Love wave device provides huge design suppleness, where the energy limitation is determined by deposited overlayer thickness and the acoustic properties. In the liquid sample, within about 60 nm from the device surface, the evanescent field of the shear acoustic wave probes’ electric, viscosity, and mass changes occurs (Figure 8b). Consequently, by monitoring the acoustic wave propagation characteristics, including the frequency, phase, and amplitude, it is probable to detect the binding kinetics and obtain the corresponding acoustic to the optical immuno-sensor [89].

### 6.8. Application of Acoustic Sensors in Tissue-Level Detection

According to Garrett and Wang [21], the acoustic sensors have significant sensitivity for tight focus inside biological tissues. As an example, the authors mentioned that a transducer with 100 MHz and an active area = 30 mm^2^ can achieve a 0.06–0.6 mPa Hz^–1^ sensitivity. It could virtually detect the chemical composition inside the biological tissues by 0.7*λ* (*λ*: acoustic wavelength). Lakshmanan, Jin, Nety, Sawyer, Lee-Gosselin, Malounda, Swift, Maresca and Shapiro [92] used acoustic biosensors for easy bioimaging of the chemicals of live tissues [115]. In that study, they used 1.2 × 1.2 mm^2^ of C57BL/6J male mice colon lumen tissues for tracking the endopeptidase enzyme concentrations that were produced by *E. coli* in the deep animal tissues with high spatiotemporal resolution (below 100 μm and 1 ms, respectively). The tissues had acoustic impedance values resulting in relatively strong reflections; the biological tissues and the wide range of their fibers, cells, and organelles affects the acoustic wave scatters. As an example, Jathoul et al. [116] developed a photoacoustic imaging system that allowed in vivo high-resolution imaging at a depth beyond that of the normal optical microscopy (10 mm in depth with a spatial resolution below 100 μm). More importantly, that system discriminated the tyrosinase enzyme composition and its genetic expressing in the live tissues of the non-vascularized invisible tissues with selective labeling of their cells. Each tissue is characterized by a different attenuation coefficient value, which increases nonlinearly with frequency [117]. As the acoustic waves pass through the tissues, the acoustic waves also deposit momentum into that tissue, resulting in mechanical forces known as acoustic radiation forces (ARF) which could be easily detected and analyzed [118].

## 7. Conclusions

Acoustic wave sensors are extremely versatile devices that are just beginning to realize their commercial potential. Meanwhile, the ASW sensors require no high operating power and preparation for monitoring of real-time cellular chemical, morphological, and functional characteristics. Other applications include measuring cell acceleration, real-time shock, viscosity, and biological media composition. It is highly evident from the above presentation that ASW techniques are in high demand and an active research area because of their potential application to different biological and chemical applications as biosensors. Even though there are a wide variety of applications of these devices with several advantages and disadvantages, there are many opportunities where improvements can be made for future applications of these sensitive devices. In the field of microfluidics, several biosensors and other chemical sensors have been used to facilitate the access to rapid and cost-effective diagnostic platforms. In addition, these sensors have an acoustoelectric sensitivity, allowing the detection of low concentrations of toxins, heavy metals, and biomarker proteins. Love, Sezawa, and Rayleigh wave sensors have been proven to be the most sensitive in general as a result of their larger energy density on the surface of the studied solid and liquid samples. Therefore, much work is continuing in developing these important sensors for their high-accuracy future applications.

## Figures and Tables

**Table 1:** Details of different applications, along with their advantages and disadvantages, in the field of acoustic devices.

No	Type	Samples	Acoustic Frequency	Active Area	Application	Advantages	Disadvantages	Ref
1	Chromium-coated QCMs	Polyclonal goat anti-rabbit IgG	5 MHz	10 mm^2^	Measurement of antigens	Simplicity, small volumes of sample, free of expensive reagents, easy fluid control in dynamic mode, and low cost.	QCM sensors have complex circuitry, poor signal-to-noise ratio, and can be influenced by humidity.	[22]
2	LiTaO_3_-based Love wave sensor	Animal stem cells	128 MHz	10 × 12 mm	Quantitative measurements of cell activities	Real-time measurement and propagation could be controlled through the used quartz crystal, allowing a simple, non-invasive, and quantitative measurement of the adherent cells’ viscoelastic properties.	Lack of experiment-related discussion, and there is little research focusing on the theoretical modeling of cell-based Love wave sensors and in-depth comprehensive theoretical analysis.	[23]
3	Piezoelectric lithium niobate (LiNbO_3_)	Pancreatic cancer diagnosis	28.3 MHz	16 × 40 mm	microRNA and oligonucleotide	Label-free, specific on-chip detection of RNA is achieved by using a separate device.	The lysis rate was only 38%. Significant improvements are needed for optimizing the time.	[24]
4	AT cut quartz substrate	Spirulina (*Arthrospira platensis*) cells	117 MHz	--	Measurement of cadmium and mercury heavy metals	The detection limit was determined to be 10^−14^ M for each metal. Not specific to a single metal, but it provides a global response to the presence of heavy metals in a trace amount.	Should be applied with other micro-organisms for achieving other toxicity tests.	[25]
5	AT-cut quartz substrate	*Escherichia coli* (*E. coli*) bacteria	118 MHz	10 mm^2^	Measurement of bacteria antigen/antibody reactions	Offer a great and specific affinity especially when a monoclonal antibody is used through detecting the specific interaction between that antibody and the antigen.	The limitation of the solid/liquid interface of the sensor with the higher biological environment to the limited sensing area of 10 mm^2^.	[26,27]
6	Quartz crystal	Bovine and pig tissues	392 MHz	25 × 2.5 mm^2^	Odorant-binding proteins	The high sensitivity of 5.63 Hz/ppm was obtained with a detection limit of 1.78 ppm with high reproducibility.	Needs low viscosities liquids for uniformly coating the active area of SAW resonators.	[28,29]
7	YX-LiTaO3 crystals	Plasma/serum	426.4 MHz	4 × 4 mm	C-reactive protein (CRP)	It successfully distinguished the human CRP serum normal concentrations from the bacterially infected tissue injury.	The limited binding ability of CRP is based on the period. In addition, especially in serum samples, the adsorption capacity was different among the samples and, consequently, signal responses were affected.	[13,30]
8	Y-cut X-rotated lithium niobate (LN)	Protein solutions	900 MHz	5 mm diameter holes	Biotin-polyethylene glycol-thiol and streptavidin	The sensitivity was 296 m^2^ kg^–1^ and the limit of detection was 104 pM. Adapted for cancer biomarker detection.	Sensitivity is still lower than those based on optical detection.	[31]

## Data Availability

Not applicable.
